# Minimal residual disease profiling predicts pathological complete response in esophageal squamous cell carcinoma

**DOI:** 10.1186/s12943-024-02006-x

**Published:** 2024-05-10

**Authors:** Pinli Yue, Fenglong Bie, Jiarun Zhu, Lin-Rui Gao, Zhendiao Zhou, Guangyu Bai, Xiaobing Wang, Ziyi Zhao, Ze-Fen Xiao, Yong Li, Aiping Zhou, Wen-Yang Liu, Yuchen Jiao, Shugeng Gao

**Affiliations:** 1 State Key Laboratory of Molecular Oncology, National Cancer Center/National Clinical Research Center for Cancer/Cancer Hospital, Chinese Academy of Medical Sciences and Peking Union Medical College, 17 Pan-jia-yuan South Ln, Chaoyang, District, Beijing, 100021 China; 2 Department of Thoracic Surgery, Shandong Provincial Hospital Affiliated to Shandong First Medical University, Jinan, Shandong 250021 China; 3 Department of Radiation Oncology, National Cancer Center/National Clinical Research Center for Cancer/Cancer Hospital, Chinese Academy of Medical Sciences and Peking Union Medical College, 17 Pan-jia-yuan South Ln, Chaoyang, District, Beijing, 100021 China; 4 Department of Thoracic Surgery, National Cancer Center/National Clinical Research Center for Cancer/Cancer Hospital, Chinese Academy of Medical Sciences and Peking Union Medical College, 17 Pan-jia-yuan South Ln, Chaoyang, District, Beijing, 100021 China; 5 Harrow International School Shenzhen Qianhai, Shenzhen, China; 6 Department of Medical Oncology, National Cancer Center/National Clinical Research Center for Cancer/Cancer Hospital, Chinese Academy of Medical Sciences and Peking Union Medical College, Beijing, China; 7 Institute of Cancer Research, Henan Academy of Innovations in Medical Science, Zhengzhou, Henan China

**Keywords:** Esophageal squamous cell carcinoma, Minimal residual disease, Pathological complete response, Neoadjuvant therapy, Adjuvant therapy, Definitive radiotherapy

## Abstract

Accurate presurgical prediction of pathological complete response (pCR) can guide treatment decisions, potentially avoiding unnecessary surgeries and improving the quality of life for cancer patients. We developed a minimal residual disease (MRD) profiling approach with enhanced sensitivity and specificity for detecting minimal tumor DNA from cell-free DNA (cfDNA). The approach was validated in two independent esophageal squamous cell carcinoma (ESCC) cohorts. In a cohort undergoing neoadjuvant, surgical, and adjuvant therapy (NAT cohort), presurgical MRD status precisely predicted pCR. All MRD-negative cases (10/10) were confirmed as pCR by pathological evaluation on the resected tissues. In contrast, MRD-positive cases included all the 27 non-pCR cases and only one pCR case (10/10 vs 1/28, *P* < 0.0001, Fisher’s exact test). In a definitive radiotherapy cohort (dRT cohort), post-dRT MRD status was closely correlated with patient prognosis. All MRD-negative patients (25/25) remained progression-free during the follow-up period, while 23 of the 26 MRD-positive patients experienced disease progression (25/25 vs 3/26, *P* < 0.0001, Fisher’s exact test; progression-free survival, *P* < 0.0001, log-rank test). The MRD profiling approach effectively predicted the ESCC patients who would achieve pCR with surgery and those likely to remain progression-free without surgery. This suggests that the cancer cells in these MRD-negative patients have been effectively eliminated and they could be suitable candidates for a watch-and-wait strategy, potentially avoiding unnecessary surgery.

**Supplementary Information:**

The online version contains supplementary material available at 10.1186/s12943-024-02006-x.

## To the editor

Advances in neoadjuvant therapy have helped a significant percentage of esophageal squamous cell carcinoma (ESCC) patients to reach pathological complete response (pCR) before surgery, creating the opportunity to modify rigorous standard-of-care regimens [1, 2]. In theory, esophagectomy, a traumatic surgery including dissection of the esophagus and stomach and anastomosis to create a tubular stomach, could be waived for pCR cases as all cancer cells in the tumor lesion have been eliminated. However, the current method to confirm pCR necessitates undergoing this radical surgery. Accurately predicting the elimination of cancer cells and pCR status before surgery could improve the management of cancer patients. By precisely identifying patients who have achieved pCR, it becomes possible to adopt a watch-and-wait approach, potentially avoiding unnecessary surgical interventions and thereby preserving the quality of life for these patients [3]. Minimal residual disease (MRD) status based on cell-free DNA (cfDNA) has been associated not only with the prediction of recurrence, but also pathological response to neoadjuvant therapy [4–7]. However, the sensitivity of current MRD profiling approaches is not sufficient to determine pCR status to waive the surgery as a significant percentage of MRD-negative cases were not pCR [4].

In this study, we developed an MRD profiling approach combining experimental and bioinformatic optimizations specifically designed to accurately predict the elimination of cancer cells in ESCC patients. The approach was validated in two independent ESCC patient cohorts: a neoadjuvant, surgical, and adjuvant therapy cohort (NAT cohort, NCT04460066) consisting of 38 patients, and a definitive radiotherapy cohort (dRT cohort, NCT05543057) including 51 advanced-stage patients. A total of 89 baseline tumor biopsies and 160 plasma samples were collected at various longitudinal time points for MRD analysis.

## Development of a pipeline for sensitive detection of MRD

To enhance the sensitivity and specificity of MRD detection, we implemented a tumor-informed, personalized assay with several key optimizations. Firstly, we significantly increased the number of profiled mutations to 40, compared to the 16–20 mutations typically monitored in prior studies [8, 9]. We selected 40 somatic mutations from the exome sequencing data of each primary tumor and tailored a personalized assay to profile these mutations in cfDNA from matched plasma samples (supplementary methods). Secondly, we used redundant sequencing and unique identifier barcodes to filter false positives caused by PCR amplification and sequencing errors [10, 11]. Additionally, we refined the mutation status by comparing mutation signals to the background noise in control cfDNA samples from individuals without the tumor, which helps exclude non-tumor derived mutations [8, 12]. For samples confirmed as MRD-positive, the ctDNA fraction was quantified based on the number and frequency of the detected mutations (supplementary methods, Fig. S1) [9].

To assess the accuracy of MRD profiling assay, we generated standard reference samples by diluting KYSE-150 cells into HEK-293T cells at varying ratios, ranging from 0 to 100% (supplementary methods). We then applied our assay to the DNA of these mixed samples, focusing on 40 single nucleotide polymorphisms unique to KYSE-150. The calculated KYSE-150 DNA fraction demonstrated a strong linear correlation with the theoretical dilution ratios, up to a ratio of 0.001% (R square = 1) (Fig. S2). The analytical validation revealed that our assay achieved 100% sensitivity (20/20) at the 0.001% KYSE-150 DNA fraction and 95% specificity (19/20) at the 0% KYSE-150 DNA fraction (Fig. S3).

## Prediction of pCR by presurgical MRD status in a neoadjuvant treatment cohort

**Fig. 1:**
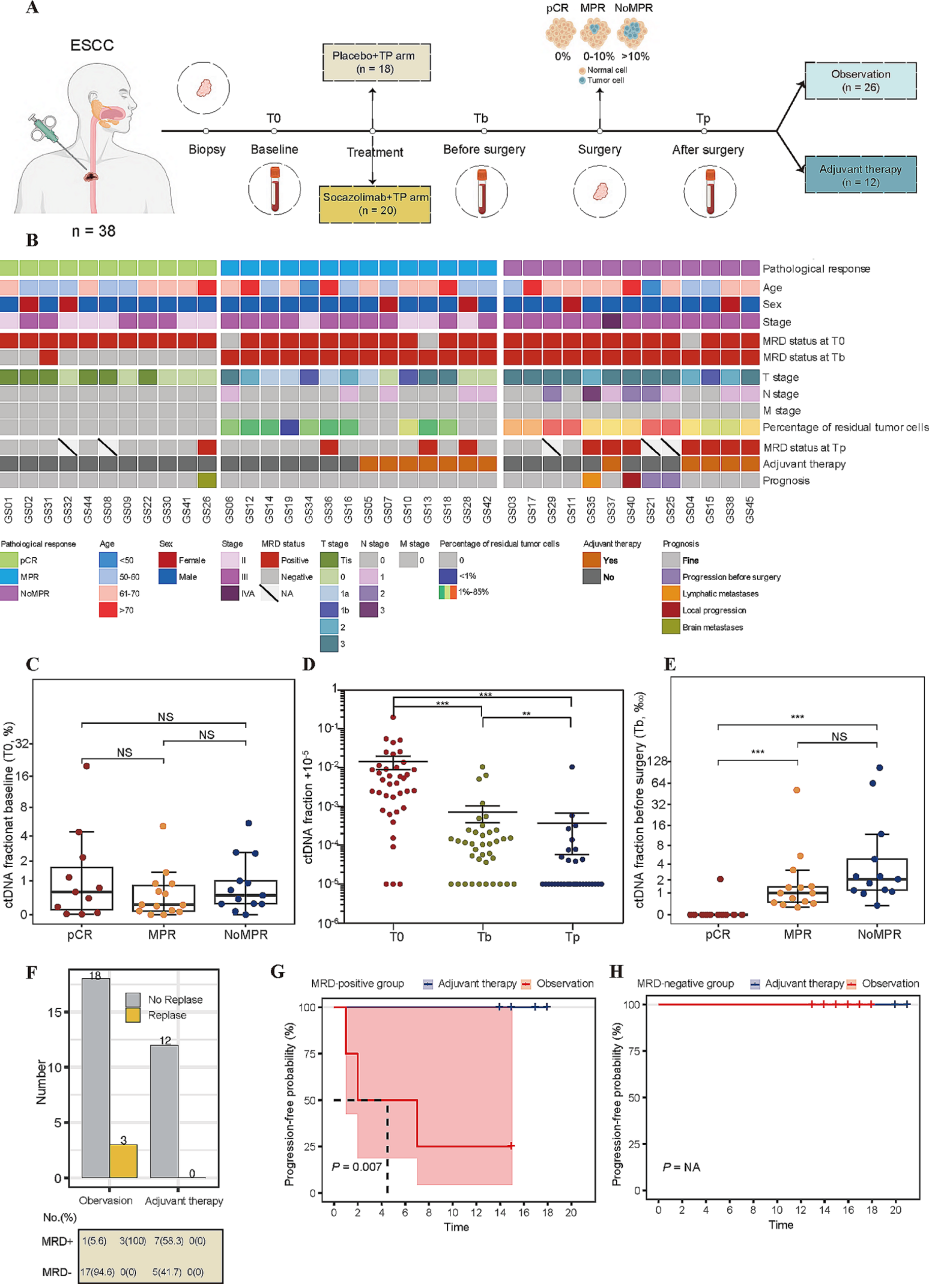
Clinical characteristics and ctDNA fractions of the neoadjuvant therapy cohort. (**A**) Study overview. Patients diagnosed with locally advanced ESCC were randomized into two groups: the socazolimab + TP arm (*n* = 20) and the placebo + TP arm (*n* = 18). Following neoadjuvant treatment, all participants underwent video-assisted thoracoscopy esophagectomy. In the socazolimab + TP arm, patients with pathological residues after surgery were administered adjuvant therapy. Tumor biopsy tissue samples were collected before neoadjuvant treatment by esophagogastroscopy. Blood was collected at three distinct phases: pretreatment (baseline, T0); before surgery (Tb); and one month after surgery (Tp). TP denotes the combination of nab-paclitaxel and cisplatin. (**B)** Patient overview, tumor characteristics, and MRD profiling outcomes. “Stage” refers to the clinical stage of patients upon enrollment. “T stage,” “N stage,” and “M stage” denote the pathological stages of the excised tumor. Percentage of residual tumor cells, pathological evaluation of the proportion of residual tumor cells in resected tumor tissue. NA, not available. (**C**) ctDNA fractions at T0 grouped by pathological responses. (**D)** ctDNA fractions of all the cases at T0, Tb, and Tp. (**E**) ctDNA fractions at Tb grouped by pathological response. (**F)** Distribution of MRD-positive and MRD-negative cases at Tp categorized on adjuvant therapy and relapse. **G** and **H**. Kaplan-Meier survival analysis depicting progression-free survival (PFS) probability based on adjuvant therapy in (**G**) MRD-positive patients and (**H**) MRD-negative patients at Tp. *P* value is not provided in (H) as none of the patients exhibited progression. NS, no significance; ** *P* < 0.01; ***P < 0.001

We validated the approach in a prospective cohort, comprising 38 ESCC patients with a comprehensive treatment regimen that includes neoadjuvant treatment, surgical intervention, and adjuvant therapy (Fig. 1A). The clinicopathological characteristics of the 38 participants are detailed in Table S1. Pretreatment tumor biopsies were collected from 38 patients, and 109 blood samples were collected at pretreatment (baseline, T0), before surgery (after neoadjuvant therapy, Tb), and 1 month after surgery (Tp). The primary endpoint was pathological response, and the secondary endpoint was progression-free survival (PFS). pCR was defined as the absence of residual tumor cells (including primary tumors and lymph nodes). No residual tumor cells or less than 10% were defined as a major pathologic response (MPR), while more than 10% were defined as NoMPR. After neoadjuvant treatment, 11 patients were identified as having a pCR based on the pathological assessment of the excised tissue. Among the remaining patients, 14 were categorized as having an MPR, while 13 showed NoMPR (Fig. 1B and S4).

We conducted exome sequencing on pretreatment tumor biopsies from the 38 patients. For each tumor, we selected 40 somatic mutations and developed a personalized assay to profile these mutations in the cfDNA with Mutation Capsule technology (Table S2). To remove target-specific background errors, we further refined the mutation status by comparing the signal in the cfDNA from the matched ESCC patient (test cfDNA) against the background noise in cfDNA samples from 10 healthy controls (control cfDNA) (Fig. S5).

In our cohort of 38 ESCC patients, 92% (35/38) had test-positive baseline plasma samples (T0), with ctDNA fractions ranging from 0.008 to 19.934% (Table S3). No significant differences were observed in baseline ctDNA fractions among the pCR, MPR, and NoMPR groups (Fig. 1C, *P* = 0.47). ctDNA fractions exhibited a substantial decline after the neoadjuvant treatment (Fig. 1D and S6, *P* < 0.001).

The pathological response in the resected tumors was closely linked to the pre-surgical MRD status (Tb). Specifically, all MRD-negative cases (*n* = 10) were pCR, and only one of the 11 pCR cases was MRD-positive at the Tb time point. Conversely, all the MPR and NoMPR cases (*n* = 27) were MRD positive (10/10 vs. 1/28, *P* < 0.0001, Fisher’s exact test; Fig. 1E). In this case, our MRD profiling assay demonstrated 100% sensitivity (27/27) and 91% specificity (10/11) in detecting residual tumor cells based on the pre-surgical blood samples.

## Utility of post-surgical MRD status for adjuvant treatment selection

We extended our personalized MRD profiling assay to blood samples collected after surgery. Overall, surgical intervention led to a significant reduction in ctDNA fractions (Fig. 1D, *P* = 0.001). In the post-surgical setting, 11 cases remained MRD-positive, while 22 cases were MRD-negative. Adjuvant treatment conferred a significant benefit for the MRD-positive cases: all seven cases receiving adjuvant therapy remained progression-free, whereas three out of the four cases under observation progressed during the median follow-up period of 17 months (Fig. S7 and 1 F-G, PFS, log-rank test *P* = 0.007, HR = 0.032, 95%CI = 0.003–0.390). Conversely, all 22 postsurgical MRD-negative cases remained progression-free, both with (*n* = 5) and without (*n* = 17) adjuvant therapy, during the follow-up period (Fig. 1H, *P* value was not available for none of the patients progressed). In this context, MRD profiling of post-surgical cfDNA effectively identified patients likely to benefit from adjuvant treatment. Without MRD-based stratification, no significant difference in PFS was observed between the adjuvant treatment and observation groups (Fig. S8A, *P* = 0.211).

We also evaluated the performance of MRD status in post-surgical samples (Tp) against pathological response (Fig. S8B). Our analysis revealed that adjuvant treatment did not offer a significant advantage in preventing disease progression for cases that were pathological positive, when the high-risk group was defined as either non-pCR (Fig. S9A; *P* = 0.166) or NoMPR (Fig. S9B; *P* = 0.176).

## Prognostic prediction in unresectable ESCC patients based on post-dRT MRD status

**Fig. 2:**
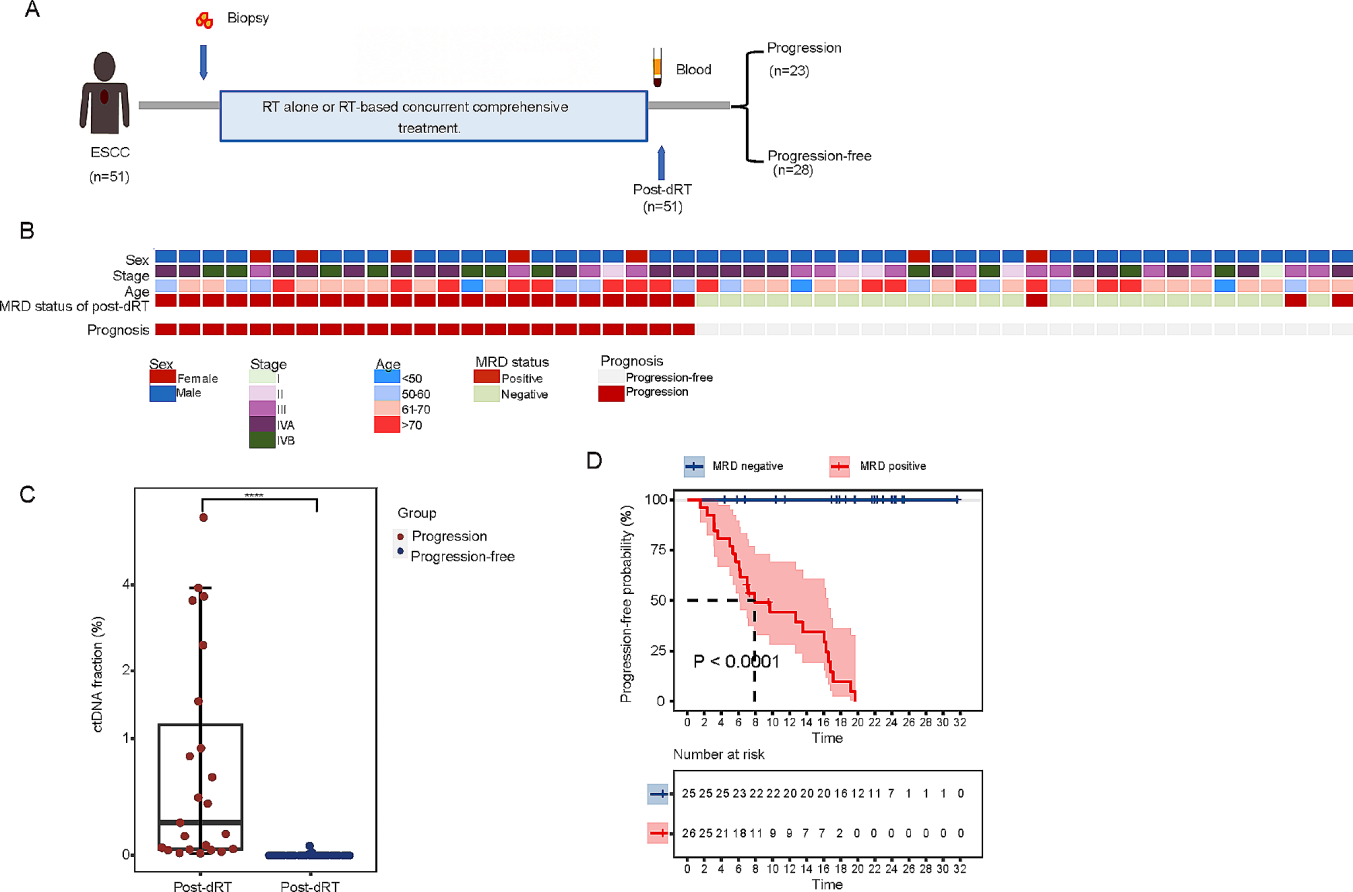
The MRD status and prognosis of definitive radiotherapy cohort. (**A)** Study design. Fifty-one patients underwent either RT alone or RT-based concurrent comprehensive treatment. Tumor biopsy samples were collected prior to treatment. Blood samples were collected after the dRT (post-dRT). RT stands for radiotherapy. (**B)** Overview of patient characteristics and MRD outcomes. Stage, clinical stage of patients at enrollment. (**C)** ctDNA fractions at post-dRT classified based on prognosis. (**D)** Kaplan-Meier survival analysis shows the probability of progression-free survival (PFS) as determined by MRD status of post-dRT. ****P < 0.0001

We extended our MRD profiling approach to a dRT cohort, in which 51 ESCC patients received either radiotherapy alone or radiotherapy-based concurrent comprehensive treatment (Table S4, Fig. 2A). None of these patients underwent surgery after dRT. Tumor biopsies were collected before treatment. Blood samples for ctDNA analysis were collected after the whole dRT process. Tumor biopsies and blood samples processing were the same to the NAT cohort.

During a median follow-up period of 19 months, 23 patients exhibited disease progression (Fig. 2B). A significant difference was observed in the ctDNA fractions between the disease progression group and the progression-free groups at the post-dRT time point (Fig. 2C, *P* < 0.0001). Of the 51 cases, 26 (51%) were MRD positive and 25 (49%) were MRD negative in the post-dRT blood (Fig. 2B). Notably, none of the MRD negative patients (0/25) showed progression, despite forgoing further surgery. Conversely, 88% (23/26) of the MRD-positive patients encountered disease progression (25/25 vs. 3/26 *P* < 0.0001, Fisher’s exact test). The post-dRT MRD status accurately forecasted prognosis by detecting residual cancer cells with a sensitivity of 100% (23/23) and a specificity of 89% (25/28). The post-dRT MRD negative patients demonstrated significantly better PFS compared to the MRD-positive patients (Fig. 2D, PFS, log-rank test *P* < 0.0001, HR = 0.043, 95%CI = 0.018–0.105).

Our study has several limitations. Firstly, the small size of our cohort may limit the generalizability of our findings, and larger clinical trials are necessary to fully validate the predictive value of MRD profiling for pCR status. Secondly, the MRD profiling assay demonstrated limited sensitivity in detecting MRD in the brain. For example, in case GS26, which developed brain metastasis one month post-surgery, the tumor cells present at the Tb time point did not release detectable ctDNA signals into circulation. This resulted in an MRD-negative result at Tb, consistent with the primary tumor’s pCR status, suggesting that additional brain scanning is necessary for patients with negative MRD results. Finally, in some cases, exome sequencing of baseline tumors might not identify enough somatic mutations for effective tracking in cfDNA, potentially compromising the assay’s sensitivity.

In this study, we aimed to develop a liquid biopsy assay capable of precisely predicting pCR before surgery. In the NAT cohort, all the presurgical MRD-negative cases by our assay were confirmed to have achieved pCR. In addition, all the post-surgical or post-dRT MRD-negative cases remained progression-free during the follow-up period in the NAT or dRT cohort. These results indicated that the MRD profiling approach was highly sensitive and had the potential to accurately identify ESCC patients in whom cancer cells have been completely eradicated. With this feature, the approach may help to provide precise management to ESCC patients by avoiding unnecessary surgery or adjuvant treatment.

### Electronic supplementary material

Below is the link to the electronic supplementary material.

Supplementary Material 1

Supplementary Material 2

Supplementary Material 3

## Data Availability

The data presented in this study are available on request from the corresponding author.

## Abbreviations

cfDNA: cell-free DNA
dRT: definitive radiotherapy
ESCC: Esophageal squamous cell carcinoma
MPR: Major pathologic response
MRD: Minimal residual disease
pCR: pathological complete response
PFS: Progression-free survival
