# Supplementary material for: Minimal residual disease profiling predicts pathological complete response in esophageal squamous cell carcinoma
Source: Mol Cancer. 2024 May 10;23:96. doi: 10.1186/s12943-024-02006-x (PMC11084057; doi:10.1186/s12943-024-02006-x)
Supplement: Supplementary file 1 — Supplementary Material 1 [file 12943_2024_2006_MOESM1_ESM.pdf]

Supplementary Figure S1. Pipeline for ctDNA detection.

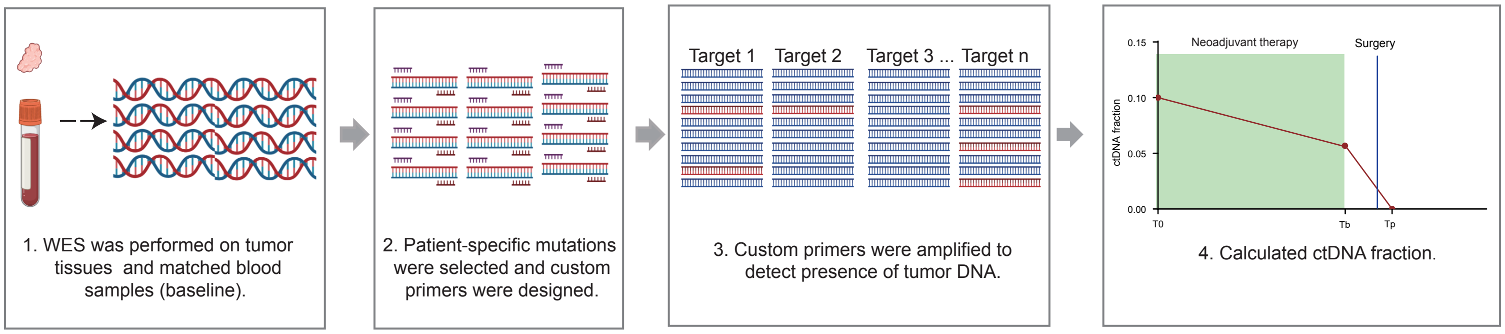

Supplementary Figure S2. The linear correlation between calculated KYSE-150 DNA fraction and theoretical dilution ratios.

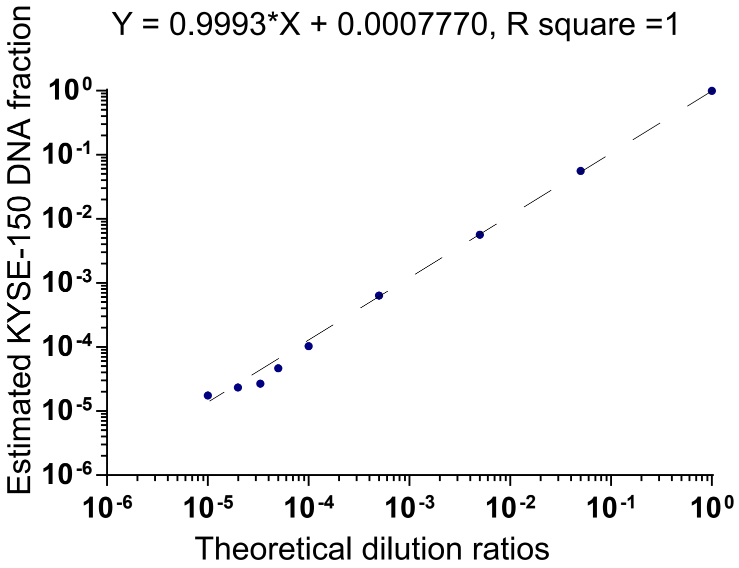

The dots had theoretical dilution ratios that ranged from 0.001% to 100%.

Supplementary Figure S3. The distribution of the detection of KYSE-150 DNA in standard reference samples.

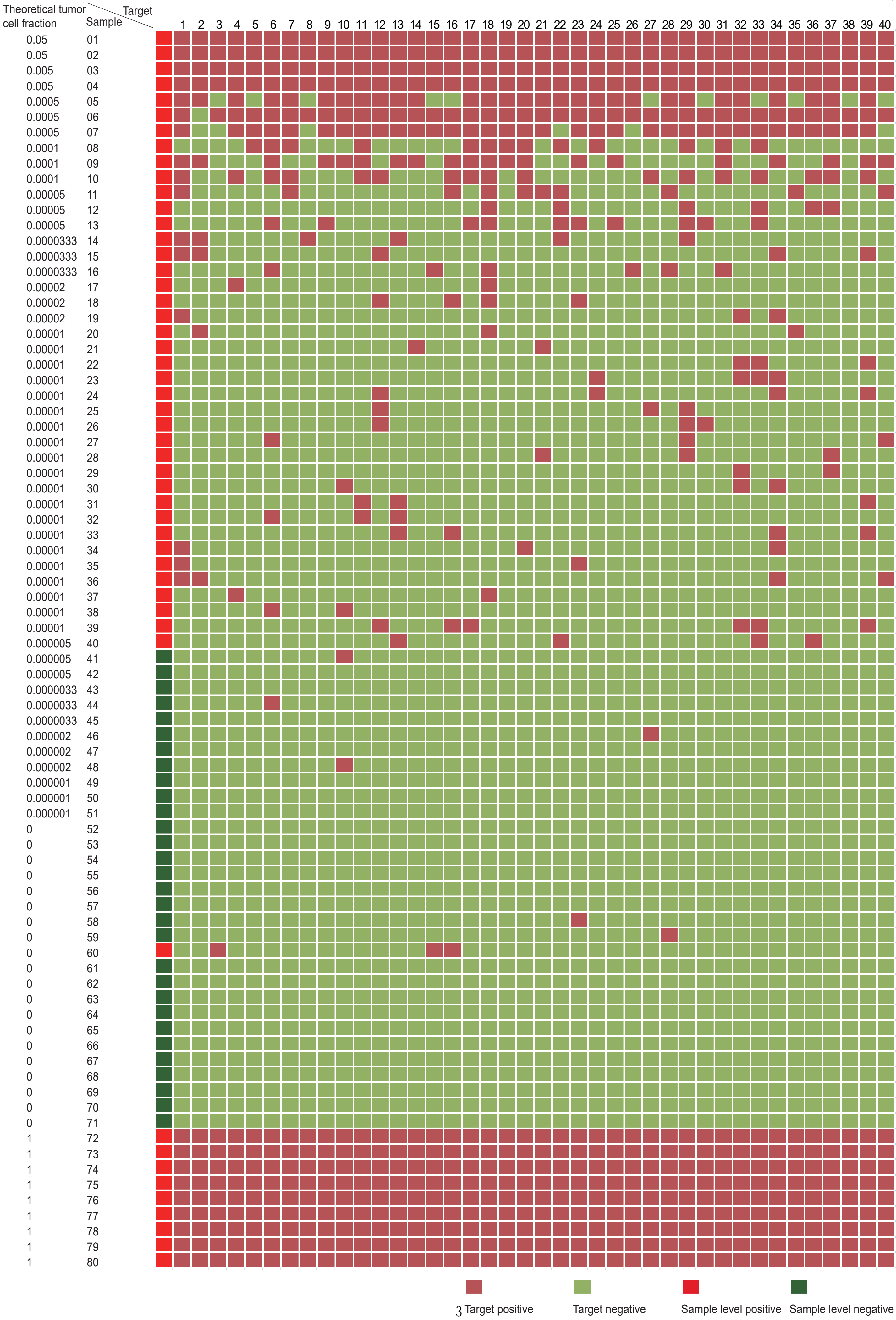

We profiled 40 target SNPs in each dilution. The samples in each dilution ratio were repeated at least twice. The samples in 0.001% and 0% dilution ratios were repeated 20 times to evaluate the limit of detection.

Supplementary Figure S4. Study design and workflow of the neoadjuvant treatment cohort.

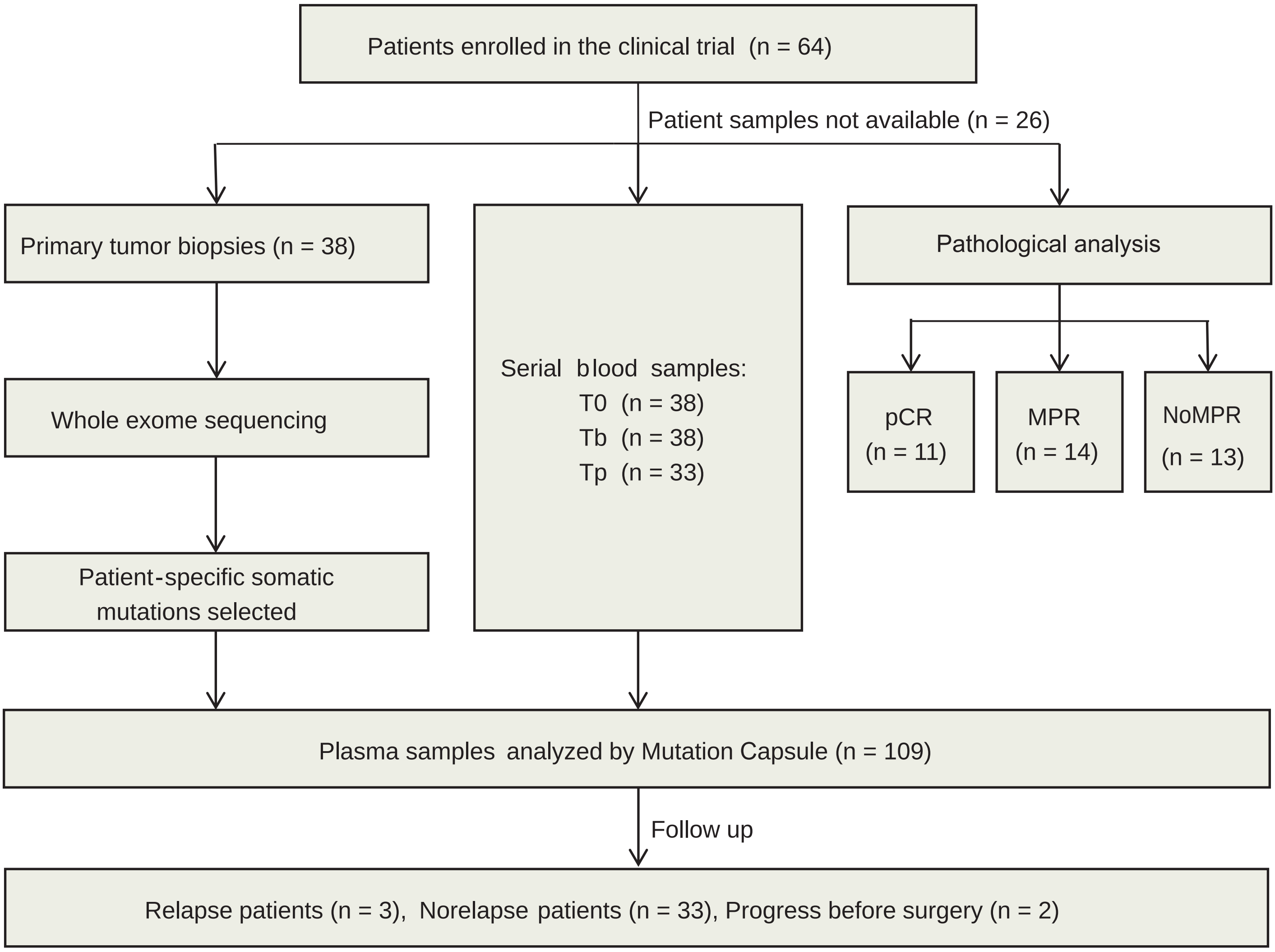

Supplementary Figure S5. The maximum mutation frequency and reads count of the background noise mutations (blue dots) identified in the cfDNA samples from healthy individuals at the sites where true mutations were identified in the cfDNA of ESCC patients.

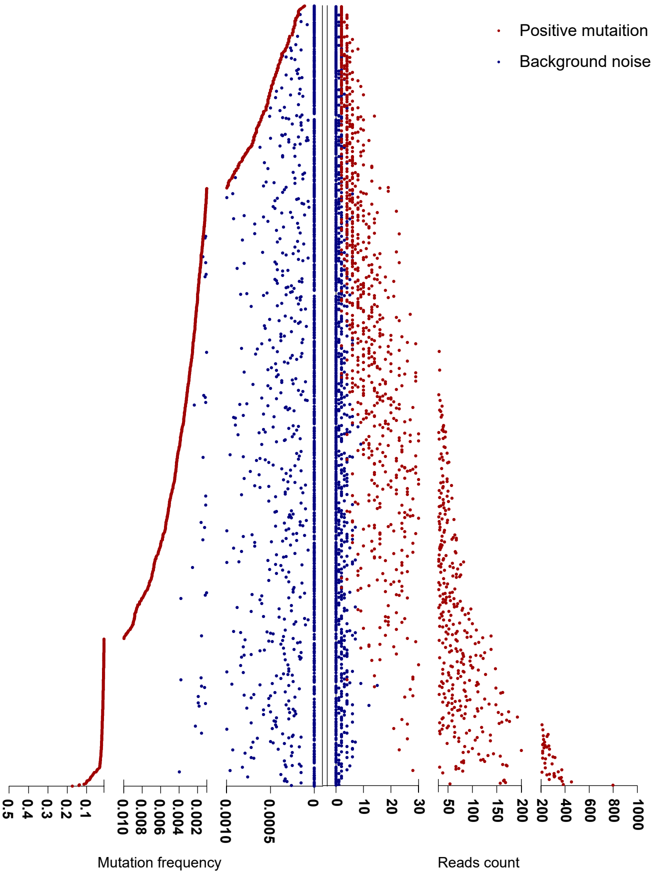

True mutations (red dots) indicate the mutations that were identified in the primary tumor and considered to be ture in the cfDNA of matched ESCC cases.

Supplementary Figure S6. ctDNA fractions in (A) pCR patients at T0, Tb, and Tp; (B) MPR patients; and (C) NoMPR patients.

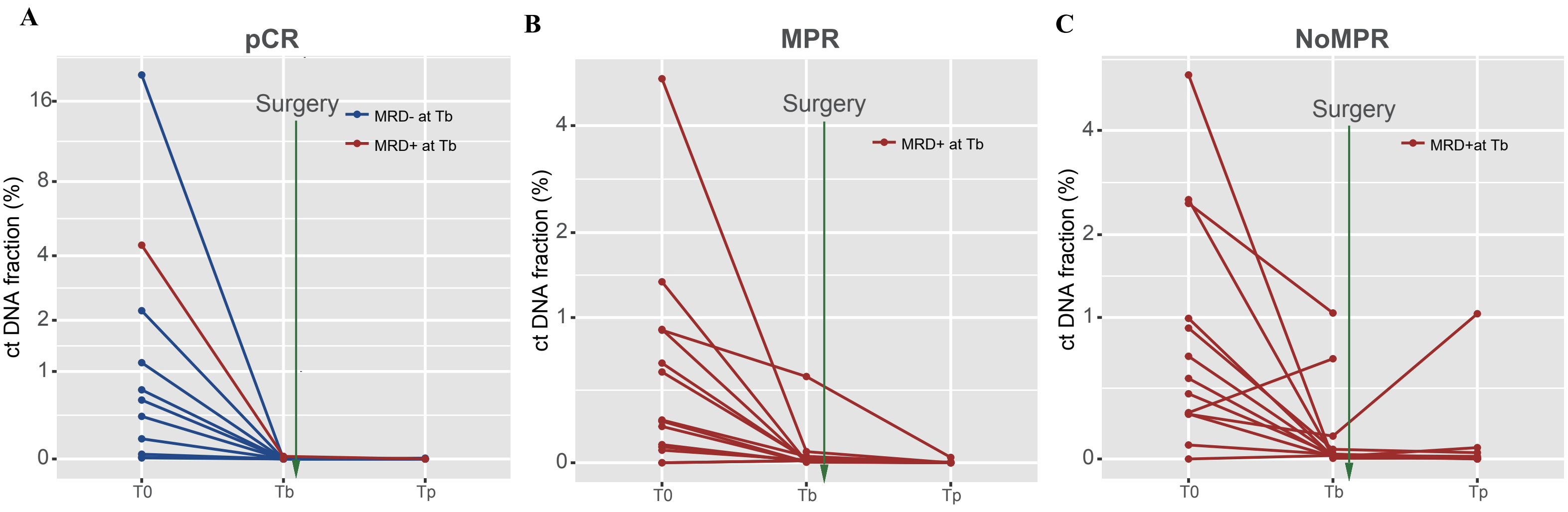

Supplementary Figure S7. Correlation between MRD status and prognosis of the neoadjuvant treatment cohort.

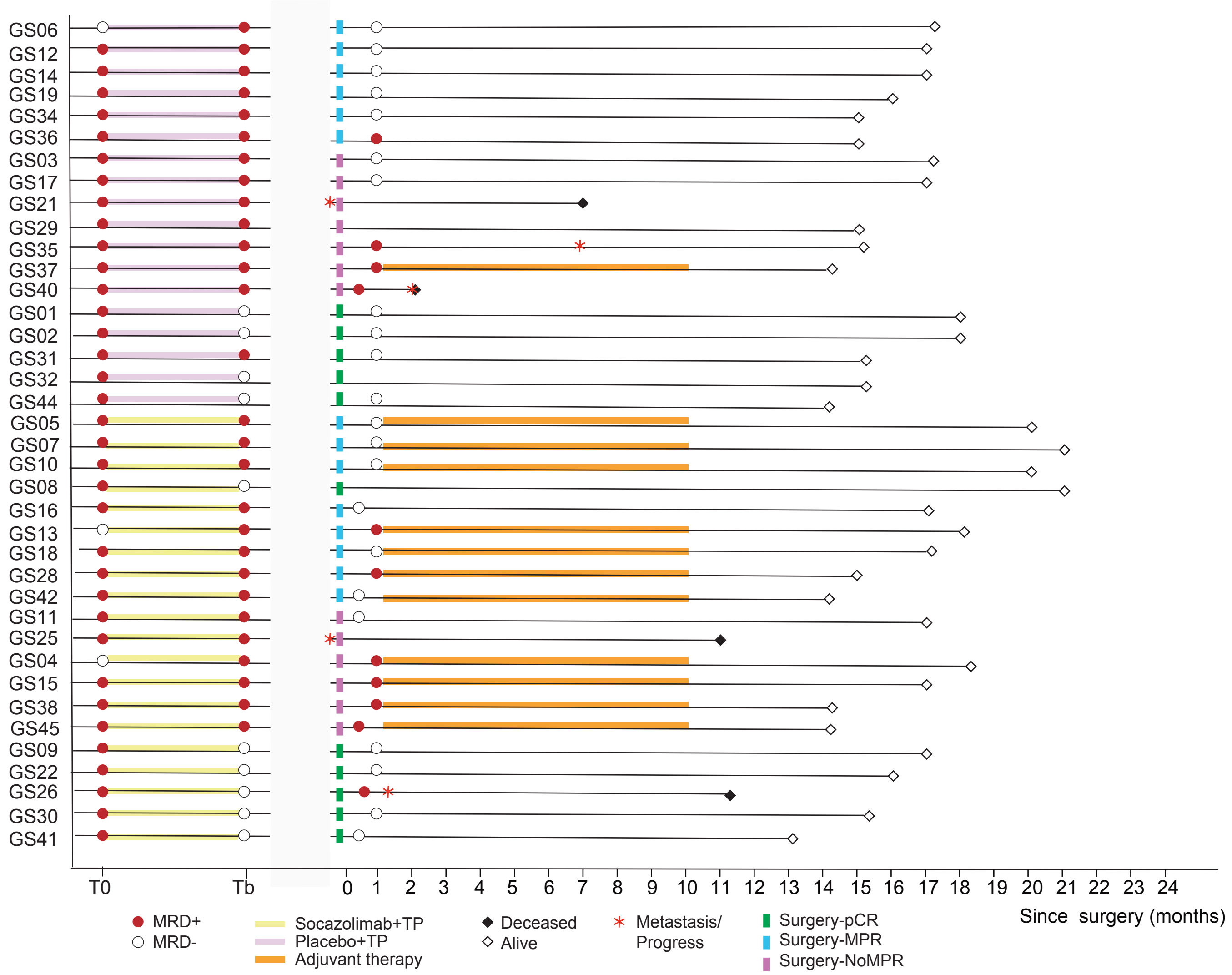

Overview of the MRD status across various time points and treatment. The right panel presents a swimmer plot illustrating the duration of follow-up and events for the 38 patients with available survival data.

Supplementary Figure S8. Association between adjuvant therapy, pathological diagnosis and prognosis.

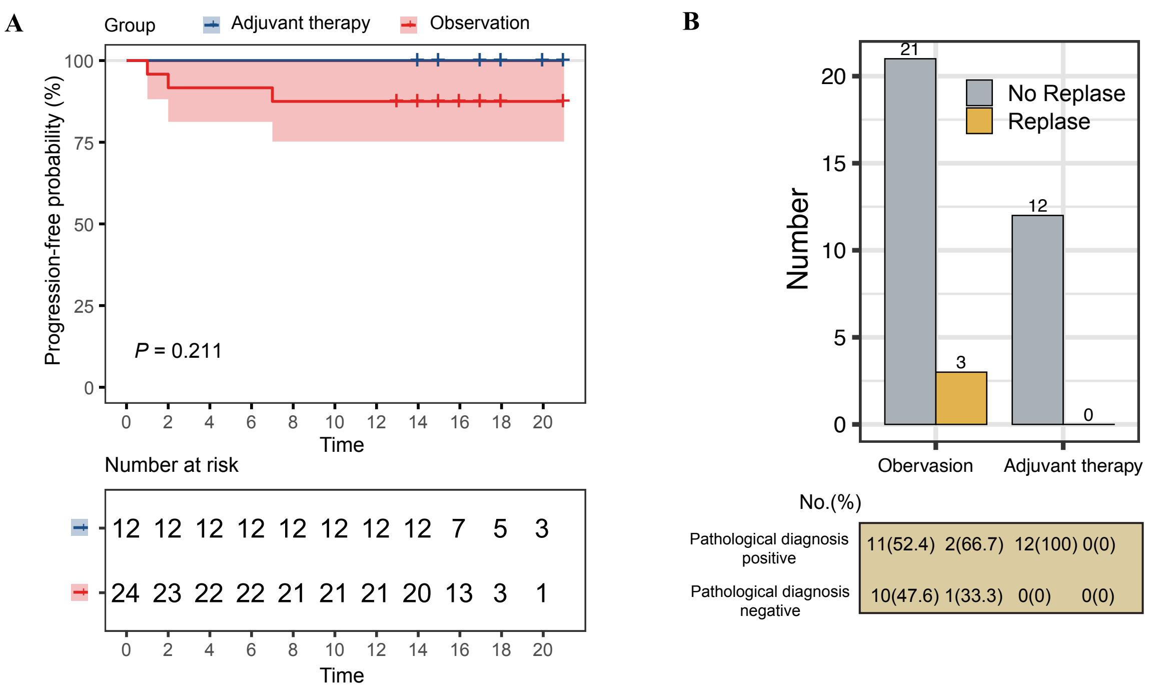

A. Kaplan-Meier survival analysis showing the probability of progression-free survival (PFS) as determined by adjuvant therapy in all patients.
B. Proportion of pathological diagnosis positive and pathological diagnosis negative patients for resected tumor based on adjuvant therapy and prognosis. Pathological diagnosis positive, MPR and NoMPR. Pathological diagnosis negative, pCR.

Supplementary Figure S9. Association between pathological response, adjuvant therapy, and prognosis.

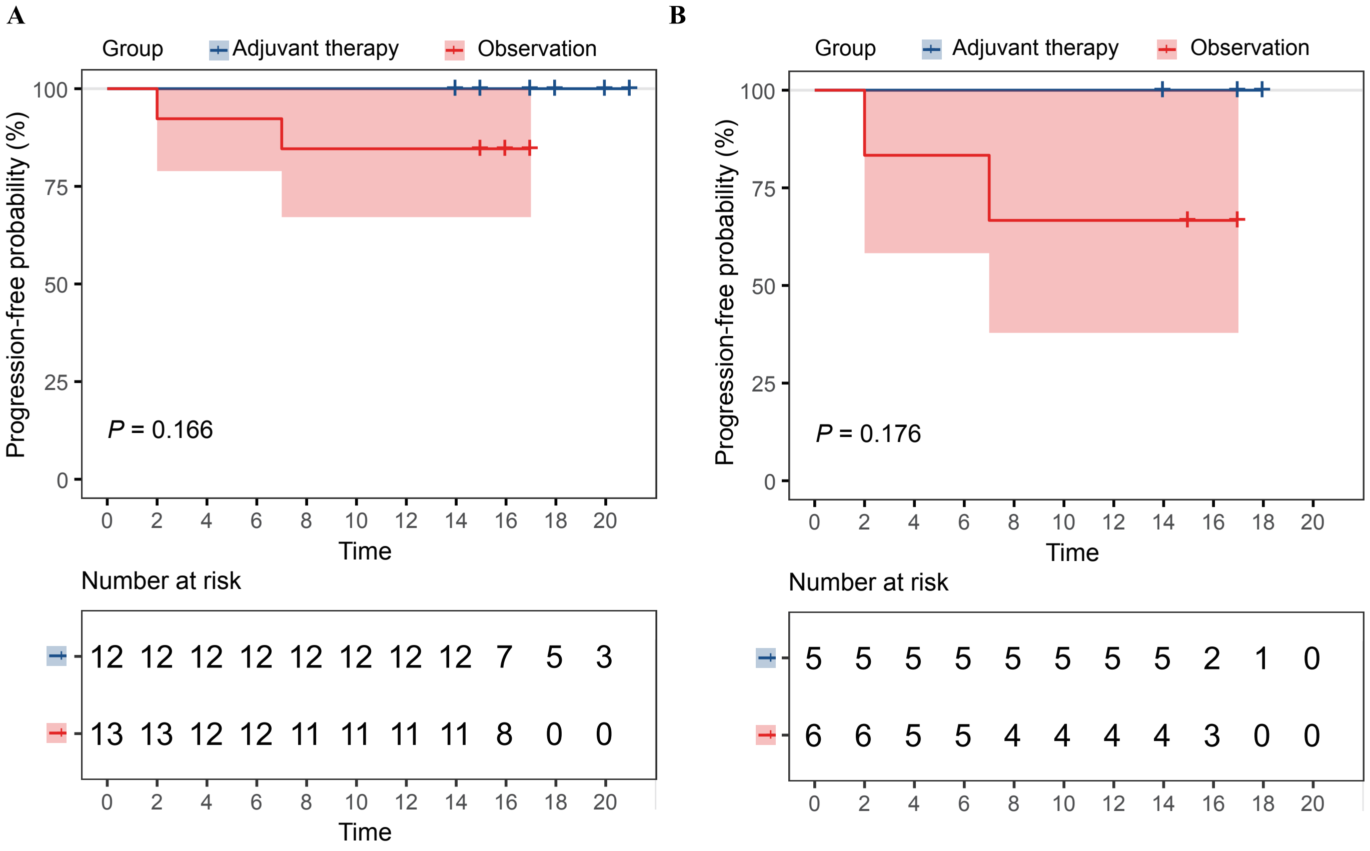

A. Kaplan-Meier survival analysis showing probability of progression-free survival (PFS) as determined by adjuvant therapy in non-pCR (MRP and NoMPR) patients; and B. in NoMPR patients.
