# Supplementary material for: Minimal residual disease profiling predicts pathological complete response in esophageal squamous cell carcinoma
Source: Mol Cancer. 2024 May 10;23:96. doi: 10.1186/s12943-024-02006-x (PMC11084057; doi:10.1186/s12943-024-02006-x)
Supplement: Supplementary file 3 — Supplementary Material 3 [file 12943_2024_2006_MOESM3_ESM.docx]

**Methods**

**Study approval**

The study was approved by the Ethics Committee of National Cancer Center, Cancer Hospital, Chinese Academy of Medical Sciences and Peking Union Medical College (approval number: 20/112-2308 and 22/036-3237). All participants granted written informed consent.

**Neoadjuvant treatment cohort**

In the neoadjuvant treatment cohort (NAT cohort), tumor biopsies and plasma samples were collected from patients with resectable locally advanced ESCC. These patients were part of a multicenter, randomized, double-blind phase II clinical trial exploring the combined neoadjuvant treatment of the PD-L1 inhibitor socazolimab and chemotherapy (NCT04460066). Eligible patients were clinically staged as T2N+M0 or T3-4aN+/-M0. Sixty-four patients were randomly assigned to two arms, receiving either socazolimab or placebo with nab-paclitaxel and cisplatin (TP), repeated every 21 days for four cycles before surgery. All patients underwent video-assisted thoracoscopy esophagectomy (McKeown) 4-6 weeks after neoadjuvant treatment. In the socazolimab + TP arm, patients with positive pathology post-surgery continued treatment with socazolimab for an additional 12 cycles or 9 months. The primary endpoint was pathological response, and the secondary endpoint was progression-free survival (PFS).

We collected samples from all patients at the National Cancer Center, Cancer Hospital, Chinese Academy of Medical Sciences research center, as samples from other research centers were not available. In total, 38 patients (59% of total participants) in the cohort were available for ctDNA analysis. Tumor biopsy tissue samples were collected before neoadjuvant treatment by esophagogastroscopy. Blood samples for ctDNA analysis were collected at the following time points: pretreatment (baseline, T0), before surgery (after the neoadjuvant treatment, Tb), and one month after surgery (Tp). Altogether, 38 tumor biopsies and 109 blood samples were collected from the 38 patients.

Blood samples were centrifuged first at 2500 rpm (1098 g) for 10 min and subsequently at 12,000 rpm for 10 min to obtain the plasma and buffy coats. Tumor biopsies, plasma, and buffy coats were stored at -80ºC until DNA extraction.

**Definitive radiotherapy cohort**

Fifty-one patients who underwent definitive radiotherapy (dRT) were included from a prospective real-world study on comprehensive treatment of ESCC (NCT05543057). All patients received volumetric intensity-modulated radiotherapy (VMAT) once daily for five days per week. The total dose ranged from 50.0 to 60.6 Gy (equivalent dose in 2-Gy [EQD2], with each fraction ranging from 1.80 to 2.14 Gy; median dose, 60.6 Gy). These patients did not have surgery after dRT.

Among the 51 patients, 8 patients exclusively underwent dRT, while 43 patients received a dRT-based comprehensive treatment (Table S4). Additionally, within the cohort, 15 patients were administered immunotherapy. Notably, for those patients with poor general conditions, rendering them unable to tolerate chemotherapy or immunotherapy, dRT alone was pursued as the preferred treatment option. Tumor biopsies were collected before treatment. Blood samples for ctDNA analysis were collected after the whole dRT process. Tumor biopsies and blood samples processing were the same to the NAT cohort.

**DNA extraction and library preparation**

Genomic DNAs were isolated from frozen tumor biopsies and buffy coats using the QIAamp DNA Mini Kit (Qiagen; Hilden, Germany). 250 ng of genomic DNA was sheared into fragments with a mean length of ~ 200 bp with the Covaris E220 instrument (Covaris; Woburn, MA, USA). The sheared DNA was prepared for whole genome library construction with the KAPA Hyper Prep Kit (Roche; Basel, Switzerland) following the manufacturer’s protocols. The genomic DNA and the yield of the libraries were quantified on the ThermoFisher Scientific Qubit 4.0 platform (ThermoFisher Scientific; Waltham, MA, USA).

**Whole-exome sequencing and identification of somatic mutations**

Agilent SureSelectXT Human All Exon V6 probe and reagents (Agilent; Santa Clara, CA, USA) were used to enrich the exome regions of tumor and matching buffy coat whole genome libraries. The enriched and amplified libraries were pooled for the Illumina Novaseq 6000 sequencing platform, and a mean depth of 200X coverage was targeted for tumor samples, and 100X coverage for buffy coats samples. The sequencing data were aligned to the hg19 reference genome using the BWA software (v0.7.17-r1188). The Genome Analysis Toolkit (GATK, v3.6), Picard (v2.7.1), and Samtools (v1.3.1) were used for basic processing, marking duplicates, local realignments, and score recalibration analysis. Sequencing data from tumor samples and matched buffy coat samples were analyzed with MuTect1 (v1.1.7) and Strelka (v1.0.14) to identify candidate somatic mutations. Integrated Genome Viewer (IGV) was used to manually inspect each mutation.

**cfDNA extraction and library preparation**

cfDNA was extracted from plasma with the Apostle MiniMax High Efficiency cfDNA Isolation Kit. For each plasma sample, a median volume of 4 mL was used. The median amount of DNA yield per plasma was 20.13 ng. cfDNA was directly used for library construction, performed with the KAPA Hyper Prep Kit following standard protocols except for the use of a customized adaptor with barcodes as previously described ^1,2^. After ligation of the adaptor, PCR amplification of the DNA fragments was performed for 10 cycles.

**Mutation profiling in cfDNA**

A tumor-informed MRD detection strategy was employed to analyze the cfDNA samples. Exome sequencing was first performed on DNA from tumor biopsies and buffy coats to obtain a list of somatic mutations for each case. For each patient, we tried to select "high quality" mutations for MRD profiling, based on features such as the confidence in IGV inspection, mutation frequency etc., for analysis in the matched cfDNA samples. We applied Mutation Capsule technology to profile the tumor-specific mutations in cfDNA ^3^. Briefly, the cfDNA sample was ligated to a customized adaptor with a random barcode as the unique identifier (UID) of the original cfDNA molecule. The ligation product was amplified for a whole genome library (MC library). A pool of customized primers was designed to amplify the target regions in the MC library targeting the 40 mutations detected in the matched tumor, and the sequencing library was sequenced on the Illumina Novaseq 6000.

The cfDNA sequencing data were processed with tag extraction (barcode sequence from the adapter) and adapter sequence deletion using Trimmomatic (v0.36), and then matched to the hg19 reference genome. Reads were grouped into UID families based on the same tags. A UID family with > 80% of reads harboring the same mutation was defined as an effective unique identifier (EUID) family for the mutation. At least 2 paired reads (reads 1 and reads 2) supporting one EUID or 2 EUIDs were required to call a mutation. All the mutations were further confirmed by manual inspection in Integrated Genome Viewer (IGV). The mutation frequency was determined by the ratio of the EUID families harboring the mutation to the sum of all UID families covering the mutation site. The details of the analysis have been described previously ^3,4^.

Furthermore, the mutation frequencies and the EUID reads counts in the cfDNA samples to be tested (test DNA) were compared with those at the same mutation sites in 10 cfDNA samples from 10 other individuals who do not harbor the tumor (control DNA). Mutations were defined as true if they met the following criteria: (1) the mutation frequency in the test DNA was higher than any of the control DNAs; and (2) the test cfDNA had at least two more EUID reads than any of the control cfDNAs. Test DNA samples with two or more mutations were defined as MRD positive.

For the MRD positive samples, the sample-level ctDNA fraction, which is the fraction of the ctDNA from tumor cells in the total cfDNA in the plasma, was evaluated with the maximum likelihood estimation based on the number of target mutations detected in the cfDNA, as well as the frequency and EUID reads of each mutation ^5^.

**Validation of the MRD profiling approach in reference samples**

To validate the performance of the MRD detection assay, two cell lines (HEK-293T and KYSE-150) were used to construct standard reference samples with serial tumor DNA fractions. The KYSE-150 cells were used as the “test cells” and were diluted with HEK-293T cells as the “control cells”. The two cell lines were counted with FACSAriaSORP flow cytometry (Becton Dickinson; San Diego, CA, USA) and serially diluted for a total of 14 dilutions (KYSE-150 cell fraction = 0%, 0.0001%, 0.0002%, 0.00033%, 0.0005%, 0.001%, 0.002%, 0.0033%, 0.005%, 0.01%, 0.05%, 0.5%, 5%, and 100%). In each dilution, the mixed cells were centrifuged to obtain a cell pellet for DNA extraction. The DNA samples were sonicated to 160-180bp in size. We performed exome sequencing on the DNA samples of pure HEK-293T and KYSE-150 cell lines to identify the SNPs in the two cell lines. Compared with the HEK-293T cell line, 40 SNPs unique to the KYSE-150 cell line were selected. The frequency of these SNPs was 100% in KYSE-150 cell line, and 0% in HEK-293T cell line. These SNPs were profiled in the DNA of the standard reference samples as “target mutations”. The 0% KYSE-150 cell fraction samples were used as the “control DNA” samples. The remaining 13 dilutions with KYSE-150 cells were considered as the “test DNA” samples.

For the standard reference samples, we used the frequencies and EUID reads counts of “target mutations” in “control DNA” samples as background noise for the 40 SNPs. The following criteria were used to determine whether a mutation in "test DNA" samples was true: (1) the mutation frequency in the "test DNA" sample was higher than any of the 10 “control DNA” samples; and (2) the "test DNA" sample had at least two more EUID reads than any of the 10 “control DNA” samples. MRD positivity and ctDNA fraction were evaluated as above.

**Pathological evaluation**

In the NAT cohort, pathological regression of the primary tumor was assessed with the Becker standard post-surgery. pCR was defined as the absence of residual tumor cells (including primary tumors and lymph nodes). No residual tumor cells or less than 10% were defined as a major pathologic response (MPR), while more than 10% were defined as NoMPR.

**Statistical analysis**

The PFS was calculated as the interval from surgery to the date of disease progression, last visit, or death, whichever occurred first. Kaplan-Meier analysis was used to plot and evaluate survival curves, and the log-rank test was applied to determine the significance of the differences between the survival curves. Statistical comparison of the ctDNA fraction in patients with pCR, MPR, and NoMPR was performed with a Kruskal-Wallis test with a significance level set at 5%. Post hoc test was done using Dunn's test with the Bonferroni correction. Statistical comparison of the ctDNA fraction at post-dRT was performed using 2-tailed Wilcoxon Mann-Whitney U test with significance level set at 5%. *P* < 0.05 was considered statistically significant.

1 Qu C, Wang Y, Wang P, et al. Detection of early-stage hepatocellular carcinoma in asymptomatic HBsAg-seropositive individuals by liquid biopsy. *Proc Natl Acad Sci U S A* 2019;**116**(13):6308-12.

2 Zhang W, He H, Zang M, et al. Genetic Features of Aflatoxin-Associated Hepatocellular Carcinoma. *Gastroenterology* 2017;**153**(1):249-62.e2.

3 Wang P, Song Q, Ren J, et al. Simultaneous analysis of mutations and methylations in circulating cell-free DNA for hepatocellular carcinoma detection. *Sci Transl Med* 2022;**14**(672):eabp8704.

4 Kinde I, Wu J, Papadopoulos N, Kinzler KW, Vogelstein B. Detection and quantification of rare mutations with massively parallel sequencing. *Proc Natl Acad Sci U S A* 2011;**108**(23):9530-5.

5 Zhao D, Yue P, Wang T, et al. Personalized analysis of minimal residual cancer cells in peritoneal lavage fluid predicts peritoneal dissemination of gastric cancer. *J Hematol Oncol* 2021;**14**(1):164.
